# Metabonomics Analysis of Stem Extracts from *Dalbergia sissoo*

**DOI:** 10.3390/molecules27061982

**Published:** 2022-03-18

**Authors:** Mengxue Li, Mengying Liu, Bingyi Wang, Lei Shi

**Affiliations:** Institute of Highland Forest Science, Chinese Academy of Forestry, Kunming 650224, China; doublemeng2019@163.com (M.L.); lmy15320577755@163.com (M.L.)

**Keywords:** *Dalbergia sissoo*, metabolomics analysis, sapwood, heartwood

## Abstract

*Dalbergia sissoo* is a woody plant with economic and medicinal value. As the pharmacological qualities and properties of the wood from this plant primarily depend on its extractives, in this study, the metabolomic analysis of extractives from its stems was carried out using UPLC-MS/MS. A total of 735 metabolites were detected from two groups of samples, heartwood and sapwood, with the largest number of terpenoids in type and the largest number of flavonoids in quantity. The PCA and cluster analysis showed significant differences in the metabolite composition between the two groups. The differential metabolites were mainly organic oxygen compounds, flavonoids, and isoflavones. Among the 105 differential metabolites, 26 metabolites were significantly higher in relative content in sapwood than in heartwood, while the other 79 metabolites were significantly higher in relative content in heartwood than in sapwood. KEGG metabolic pathway enrichment analysis showed that these differential metabolites were mainly enriched in three metabolic pathways: Flavonoid biosynthesis, isoflavonoid biosynthesis, and flavonoid and flavonol biosynthesis. This study provides a reference for metabolomics studies in *Dalbergia* and other woody plants.

## 1. Introduction

*Dalbergia sissoo* Roxb. ex DC, vernacularly known as ‘Shisham’ in Hindi, is a deciduous rosewood tree, also known as sisu. *Dalbergia* is a large genus of small to medium-size trees, shrubs, and lianas in the Leguminosae family. Shisham is widely distributed in the Indian subcontinent [1], Nepal, Pakistan, Bangladesh, and other countries, and was first introduced to Yunnan, China in 1999 [2,3,4]. Shisham, an Indian variety, is basically the same as *Dalbergia odorifera* in heartwood color, texture, and other aspects [5,6]. Its yellowish-brown wood is hard and resistant to cracking, with an aromatic smell. Additionally, the wood shows excellent qualities in termite resistance and abrasion resistance. Therefore, it is suitable for use in carving, processing, decoration, furniture, etc. [7]. In addition, it has many medicinal values, being used as an abortifacient, aphrodisiac, dewormer, febrifuge, and expectorant [8]. Previous studies regarding its phytochemical composition and clinical and pharmacological aspects have shown that Shisham has many pharmacological properties [9].

When trees grow to a certain age, heartwood forms in the interior of the xylem. However, the mechanism of heartwood formation has not been determined yet, and two widely accepted hypotheses involve the physiological function transformation of parenchyma cells and the accumulation of extracts [10,11]. The transformation from sapwood to heartwood is an extremely complex process that leads to a series of physiological and biochemical changes. Previous studies have carried out extensive investigations on numerous topics, including the programmed death of parenchyma cells, the shift in cell moisture content, the generation and accumulation of heartwood components, the change in respiratory gas content, the movement of mineral elements, and the change in non-structural carbohydrates; however, the transformation mechanism is still unclear [12,13,14,15,16,17]. In most angiosperms, ray parenchyma cells survive to the innermost sapwood tissue and show a sudden decrease in the transition zone. There are many theories regarding the actual causes of parenchymal cell death (the formation of tylosis, gums, or extractives; a decrease in moisture content; increase in gas volume, by an accelerated increase in CO_2_; phytohormones). However, these theories are not yet conclusive [18,19]. The disintegration of parenchyma cells is accompanied by the consumption of reserve substances, starch, non-structural carbohydrates, and triglycerides. Both energy and small molecules produced during degradation make important contributions, with the former supplying energy for cellular metabolism and the latter serving as precursors for extracts such as some phenolics. “Heartwood formation, a kind of programmed cell death in the oldest sapwood issues, is the final step in the life cycle of living xylem cells”. This means that an indicator of heartwood formation is the death of parenchyma cells [20]. As the end of parenchyma cells always involves the biosynthesis of extracts, the formation of heartwood is always accompanied by the continuous synthesis and accumulation of secondary metabolites [21].

Recently, the formation and accumulation of heartwood components have been a hot topic in heartwood formation research, and it is also the most important aspect of heartwood formation. These heartwood components are mostly extractives or secretions produced in the metabolism and degradation products of some tissues [22]. They endow heartwood with a unique appearance and act as potential bioprotectants, exhibiting resistance to pathogenic fungal attacks [23]. The quality and quantity of these extracts depend on species, genetic determination, and environmental control [24].

Metabolomic analysis is a research method based on the high-throughput chemical detection of data in small-molecule metabolites throughout an organism. Differential metabolites are detected and screened from organisms and used to study the corresponding metabolic processes and patterns of change. Metabolomics is now widely used in biology, medicine, pharmacology, agronomy, food, and other scientific fields [25]. It is mainly used in the study of mechanisms of the plant stress response, the diagnosis of diseases and the identification of drugs, and the identification of nutrient compositions and the quality of food [26,27]. Recently, Qiu et al. performed a large-scale targeted metabolomic analysis of two leaf tissues from nine tea varieties from five representative geographical origins in China using liquid chromatography-mass spectrometry (LC-MS). The results indicated the importance of phenylpropanoid and flavonoid biosynthetic pathways in the improvement of tea quality. Accessible resources were provided for further studies on natural metabolic changes in tea trees [28]. Shen et al. studied the effects of aluminum stress on peanut roots based on widely targeted metabolomics using ultrahigh-performance liquid chromatography-tandem mass spectrometry (UPLC-MS/MS). The results showed significant alterations in the root metabolites of peanut seedlings under aluminum stress, and the differential metabolites were mainly enriched in the isoflavone biosynthetic metabolic pathway. The study provided a theoretical basis for the effective reduction in aluminum toxicity in peanut production [29]. To date, among the various analytical techniques commonly used in metabolomics, the UPLC-MS/MS analytical method is currently one of the most widely used for standardization purposes. Its advantages include high sensitivity, no derivatization, and short analysis time; due to these, it is now widely used in standardization studies regarding various phytochemicals. Therefore, it is necessary to systematically and comprehensively study the differences in the chemical composition of heartwood and sapwood of Shisham so as to provide a reference for future research on the formation mechanism of heartwood. We studied the phytochemistry and metabolism of the most critical flavonoids in the methanol extracts of Shisham based on UPLC-MS/MS. The sapwood and heartwood samples were extracted with methanol via low-temperature ultrasound. We developed a metabonomic analytical method that included the correlation between the extracts of the sapwood and heartwood of Shisham via principal component analysis.

The value and significance of data are as follows:

- These data are helpful in determining and quantifying the types and concentrations of, and differences between, metabolites in two parts of the stem of *Dalbergia sissoo*.
- The distribution of these quantitative data can provide references for the metabonomics study of other species of *Dalbergia*.
- The correlation and database of metabolites of this species were obtained for metabonomics study in trees of medical importance.
- At present, the metabonomics of the Shisham has not been studied, so it is of great significance in the fields of biochemistry, biosynthesis, plant physiology, plant biotechnology, plant chemistry, and food chemistry.

## 2. Results

### 2.1. Analysis and Processing Results of Original Data

#### 2.1.1. Total Ion Chromatographic Results

**Figure 1:**
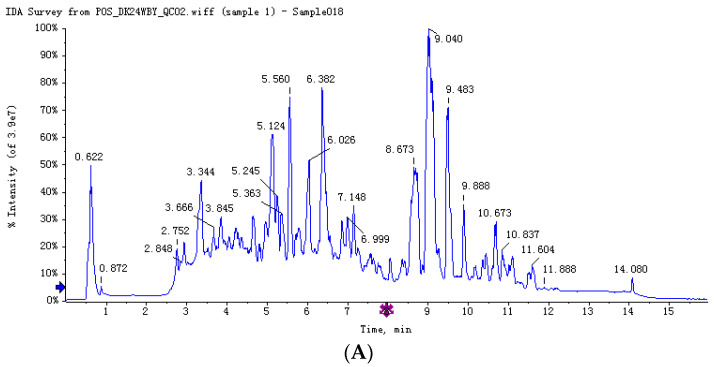

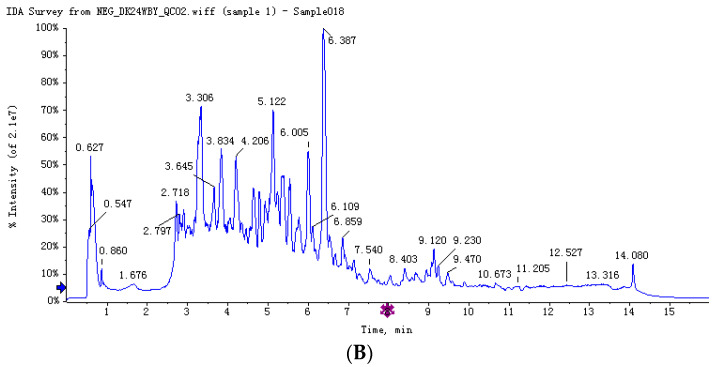
The total ion chromatograms of quality-control samples in positive and negative ion modes. (**A**) Total ion chromatographic in cationic mode (positive ion); (**B**) total ion chromatographic in anionic mode (negative ion).

After chromatographic separation, the components from the sample continuously entered the mass spectrum, and the mass spectrum was continually scanned for data collection. Each scan obtained a mass range, and the total ion current intensity was obtained by adding up all of the ion strengths in each mass spectrum. The total ion chromatogram was drawn with time as the abscissa and the sum of ion strength as ordinate. Figure 1A,B shows the total ion chromatograms of quality-control samples in positive and negative ion modes, respectively. It can be seen from the figure that the peak shape is good, and the distribution is relatively uniform under this detection condition. According to statistics (Table 1), 5704 peaks were detected in the positive ion mode and 4894 were detected in the negative ion mode (Tables S1 and S2).

#### 2.1.2. Identification Results of Metabolites

**Figure 2:**
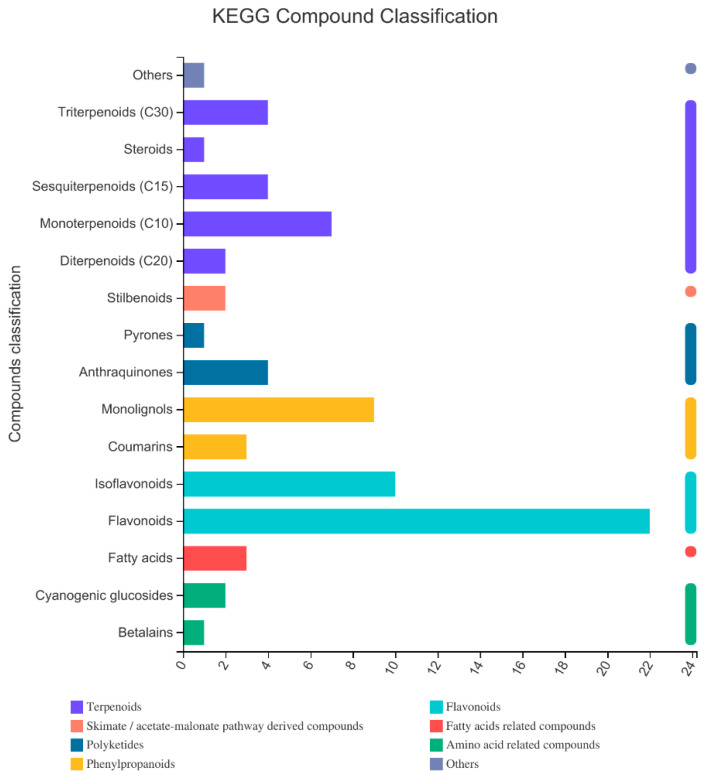
KEGG classification results of compounds. The ordinate is the classification of KEGG compounds, and the abscissa is the number of compounds annotated to this category. The bar’s color indicates that the compound belongs to the primary classification category.

Through the retrieval of self-built databases, Metlin, HMDB, and other databases, a total of 735 excellent metabolites were detected (Table S3). The number of metabolites annotated to HMDB and lipid maps was 676. The number of metabolites in the KEGG database was 182. According to the KEGG classification (Figure 2) of phytochemical compounds, the identified compounds were classified as terpenoids, polyketides, phenylpropanoids, flavonoids, fatty-acid-related compounds, amino-acid-related compounds, and others. Among them, terpenoids are the most diverse, while the largest number was observed for flavonoids.

#### 2.1.3. Correlation Analysis between Groups

**Figure 3:**
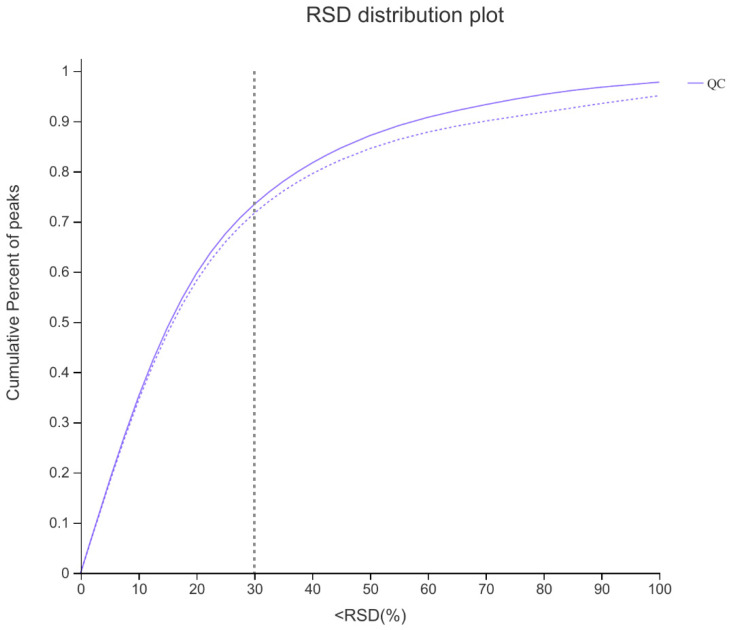
Sample evaluation. The abscissa is the RSD (%) value, that is, the standard deviation/mean value and the ordinate is the proportion of ion peak.

In order to improve the reliability of metabolic difference analysis, the metabolic data of different treatments were statistically analyzed (Figure 3). From the QC sample evaluation chart, we can see that for the overall data, when the RSD is less than 0.3, the proportion of the peak is more than 70%, so it was proven as reliable.

**Figure 4:**
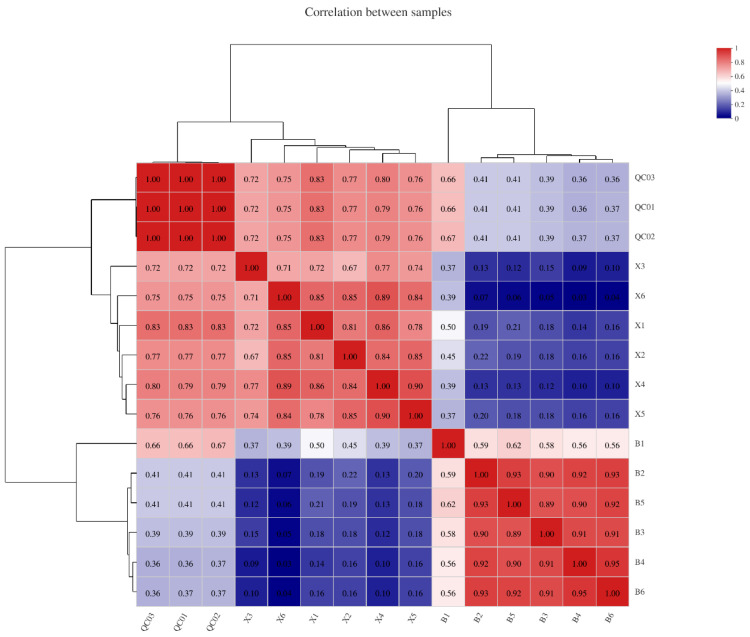
Correlation between samples. Each grid in the figure represents the correlation between two samples; different colors represent the relative size of correlation coefficients between samples; the length of clustering branches represents the relative distance between samples.

The results of the hierarchical cluster analysis showed that the two groups of samples were divided into two clusters, and there were no cross phenomena between each group. The results illustrated a significant difference between group X and group B (group X was made up of the six samples of heartwood of Shisham, including X1, X2, X3, X4, X5, and X6; group B was made up of the six samples of sapwood, including B1, B2, B3, B4, B5, and B6) (Figure 4). In the two groups, the correlation between samples in group X was more than 0.7, while in group B, except for B1, the correlation between samples was as high as 0.9. The correlation between the two groups was generally low; the highest was the correlation between X1 and B1, which was 0.5.

### 2.2. Difference Analysis of Metabonomics between Heartwood and Sapwood

#### 2.2.1. Principal Component Analysis (PCA)

**Figure 5:**
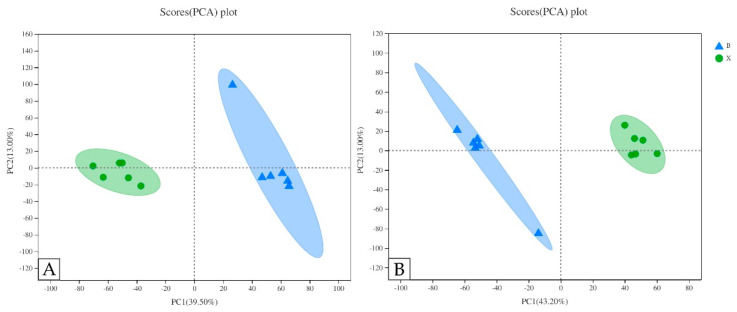
PCA score plots of different samples. (**A**) PCA score plots of two groups in cationic mode (positive ion). (**B**) PCA score plots of two groups in anionic mode (negative ion). The confidence ellipse indicates that the “true” samples of this group are distributed within this region at 95% confidence level; beyond this region, the samples are considered as possible outliers.

Firstly, the PCA model that fit the two principal components was obtained using principal component analysis (Figure 5). The distance between each coordinate point represents the degree of aggregation and dispersion between samples. The shorter the distance is, the higher the similarity between samples is. The longer the distance is, the greater the difference between samples is. The cumulative r2x was 0.994 in the anionic mode and 0.920 in the cationic mode. The parameters of the two models were greater than 0.5, and there was no significant difference between them, which indicated that the PCA model was stable and could be used for metabolic difference analysis. The PCA showed that the samples of group X and group B (group X was made up of the six samples of heartwood and group B was made up of the six samples of sapwood) were each gathered in one place, and the degree of dispersion among groups was large, which indicated that the metabolic differences among groups were large, and the repeatability of parallel samples in the group was good. The PCA showed that heartwood and sapwood samples were located in the positive and negative half axes, respectively, in the anionic or cationic mode, and they were obviously separated, indicating that there were significant differences in the composition of metabolites between the two groups.

#### 2.2.2. Orthogonal Partial Least Squares Discriminant Analysis (OPLS-DA)

**Figure 6:**
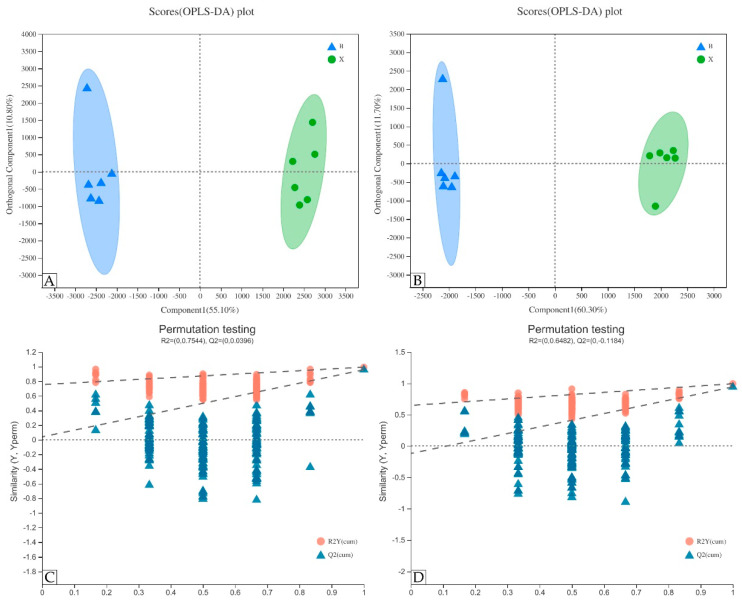
OPLS-DA model and permutation testing. (**A**,**B**) OPLS-DA scores plot in cationic and anion mode; (**C**,**D**) verification of OPLS-DA model of (**A**,**B**), respectively.

The disadvantage of PCA is that it cannot ignore the intra-group error and eliminate the random error irrelevant to the research purpose, so it is an unsupervised analysis method. In order to show the metabolic differences between heartwood and sapwood more accurately, the supervised OPLS-DA model was used for further analysis. As shown in the OPLS-DA chart, sapwood samples were mainly distributed on the left side of the confidence interval, and heartwood samples were mainly distributed on the right side of the confidence interval, which shows that the model can effectively distinguish two groups of samples (Figure 6A,B). The OPLS-DA model had two principal components: rx2 = 0.72, ry2 = 0.995, and Q2 = 0.943 in anion mode and rx2 = 0.66, ry2 = 0.993, and Q2 = 0.958 in cation mode. The parameters of these three models were all greater than 0.5, which indicates that the OPLS-DA model established in this study could effectively explain the metabolic differences between the two groups. Permutation validation can effectively evaluate whether a test model has been fitted and evaluate the statistical significance of a model by randomly changing the order of the classification variable y and establishing the corresponding OPLS-DA model many times to obtain the R2 and Q2 values of the random model. The permutation test results of the OPLS-DA model used in this study are shown in the permutation test chart (Figure 6C,D). The original R2 point on the far right is always higher than the R2 point on the left, and the original Q2 point on the right is always higher than the Q2 point on the left, indicating that there was no overfitting phenomenon in the OPLS-DA model, and the model was stable, which can better explain the differences between the two groups of samples.

#### 2.2.3. Determination and Classification of Differential Metabolites between the Two Groups

**Figure 7:**
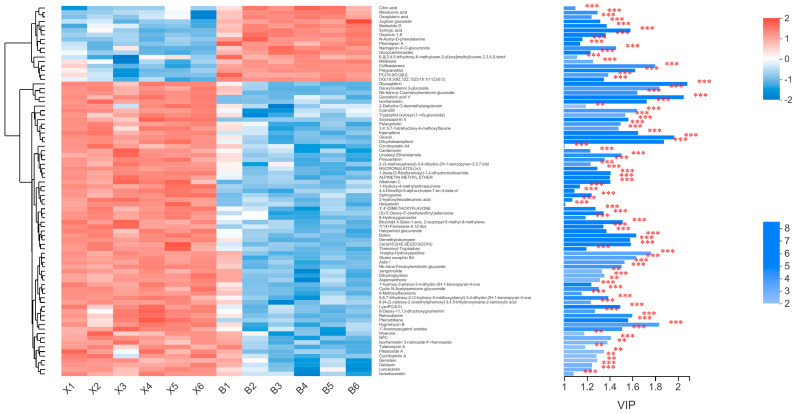
Expression profile and VIP of metabolites. Each column represents one sample, and the sample name is shown below. Each row represents a metabolite, and the color indicates the size of the relative expression of that metabolite in the sample. The correspondence between the color gradient and the numerical size is shown in the gradient color block. The right side is the metabolite VIP bar graph; the bar length indicates the contribution of the metabolite to the difference between the two groups. The default value is not less than 1, and the larger the value, the greater the difference between the two groups. The bar color indicates the significance of the metabolite difference in the two groups of samples, i.e., the *p*-value, the smaller the *p*-value, the larger the -log10 (*p*-value), and the darker the color. Right side ** represents *p* < 0.01, and *** represents *p* < 0.001.

The VIP score (Figure 7) of the OPLS-DA model (VIP >1) and the *p*-value of the *t*-test (*p* < 0.05) were used to find the differential metabolites between the two groups. A total of 105 differential metabolites were screened out from the samples of two groups (group X vs. group B). The relative contents of 26 metabolites in sapwood were significantly higher than those in heartwood, while relative contents of the others in heartwood were significantly higher than those in sapwood. Through the comparison of differential metabolites, it was found that primary metabolic products, such as citric acid, oxoglutaric acid, *N*-acetyl-d-phenylalanine, and naringenin 4′-*O*-glucuronide, were mostly enriched in sapwood in the cluster analysis diagram. Secondary metabolites such as kaempferol and pelargonidin, which are located downstream of the metabolism, were mainly concentrated in heartwood.

### 2.3. Analysis of Differential Metabolites

#### 2.3.1. Differential Metabolite Classification and Pathway Analysis

**Figure 8:**
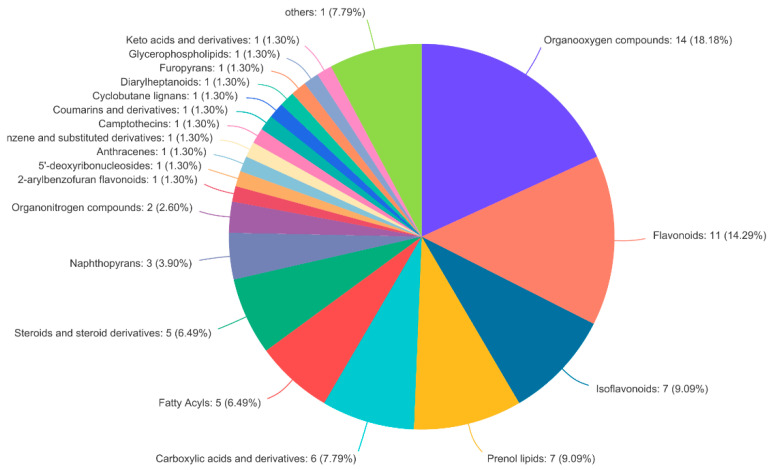
Classification of differential metabolites of HMDB.

The 105 differential metabolites between the two groups were classified using HMDB (class level). Among them, there were 14 kinds of organic oxygen compounds, 11 kinds of flavonoids, 7 kinds of isoflavones, and 7 kinds of allyl alcohol lipids. The identification results of specific components are shown in Figure 8.

The KEGG database was used to find all of the differential metabolites using KEGG IDs, and these IDs were input into the metabolome analysis database. Among 105 differential metabolites between the two groups, 23 were in the metabolic pathway. Through metabolic enrichment, the possible differential metabolic pathways of the two groups were identified, and these metabolic pathways were screened. Finally, the pathway with the highest correlation with the difference of metabolites was obtained. The results are shown in Table 2; as shown, KEGG enrichment analysis revealed three critical metabolic pathways, including flavonoid biosynthesis, isoflavonoid biosynthesis, and flavone and flavonol biosynthesis.

#### 2.3.2. Differences between Two Groups in Important Metabolic Pathways

**Figure 9:**
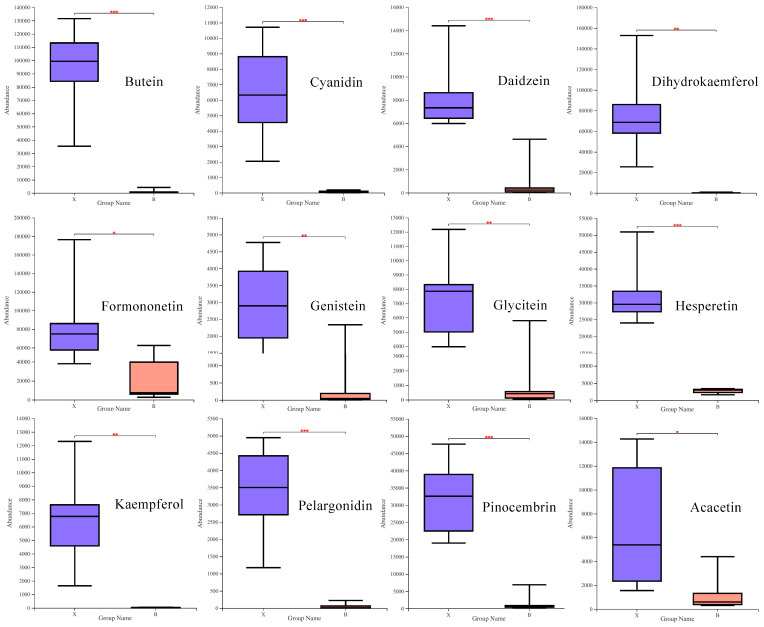
The relative contents of major differential metabolites. Groups X and B indicate the heartwood and sapwood groups, respectively. The line in the middle of the box represents the median relative expression abundance of the metabolite, and the upper and lower edges represent the maximum and minimum values of that set of data. Upper side * represents *p* < 0.05, ** represents *p* < 0.01, and *** represents *p* < 0.001.

Among the three important pathways obtained using KEGG enrichment analysis, the abundance of differential metabolites in heartwood was higher than that in the sapwood. Figure 9 shows the difference in the relative content of the main metabolites involved in the metabolic pathway between the two groups. As can be seen from the figure, the relative contents of the 12 differential metabolites listed are extremely low in sapwood, except for formononetin. By analyzing formononetin, it was found that it belongs to the isoflavonoids class and participates in the isoflavonoid biosynthesis metabolic pathway, which is the next step of daidzein.

#### 2.3.3. Topological Analysis of Metabolic Pathways

**Figure 10:**
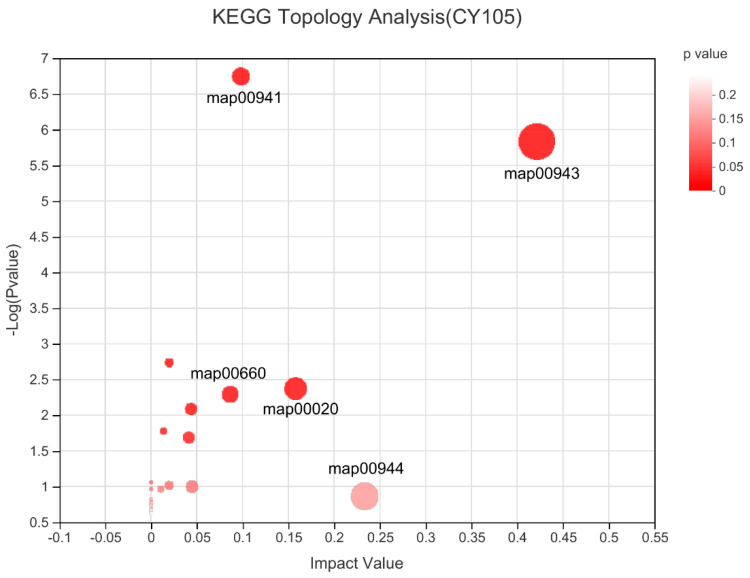
KEGG topology analysis of bubble graphs. Each bubble represents a KEGG pathway; the horizontal axis represents the relative importance impact value of metabolites in the pathway. The vertical axis represents the enrichment significance of the metabolite pathway -log10 (*p*-value).

KEGG topology analysis was performed on the metabolic pathways of differential metabolite enrichment, and the results are shown in the bubble diagram below (Figure 10). The size of the bubbles in the figure represents the impact value. The larger the bubble, the greater the importance of the pathway, which is the basis for screening metabolic pathways. Among them, the five labeled metabolic pathways all showed lower *p*-values and higher influencing factors of the pathways, and the influencing factors of the five metabolic pathways were all higher than 0.1, indicating that these five pathways played an essential role in the difference between heartwood and sapwood groups, which include map00943 (isoflavonoid biosynthesis), map00941 (flavonoid biosynthesis), map00020 (citrate cycle (TCA Cycle)), map00944 (flavone and flavonol biosynthesis), and map00660 (C5-basic acid metabolism).

## 3. Discussion

With the development of high-throughput sequencing technology, metabolomic analysis based on various chromatography-mass spectrometry tandem techniques has become increasingly mature and widely used in the metabolomic analysis of various organisms. Previous studies on Shisham have mostly focused on wood applications and medicinal values, while relatively little research has been carried out on the isolation and identification of its extracts. In this study, the metabolomic analysis of extracts from Shisham stems based on UPLC-MS/MS was carried out to lay the foundation for obtaining valuable information on the components of the wood and its subsequent isolation and utilization. Combining the experimental results and relevant references, we first discuss the types of wood extracts and their effects on wood properties and then discuss the accumulation of differential metabolites.

### 3.1. The Importance of Wood Extracts

Extractives account for a small portion of wood, but greatly influence the properties of wood, for instance, in the permeability of wood, corrosion resistance, color, and other aspects [30]. *Dalbergia sissoo* is a precious rosewood, in which the anatomical structure and color of heartwood and sapwood are obviously different. Despite this, the differences in extracts from different parts of its wood have not been reported [31]. The clustering heat map and PCA analyses of metabolites showed that there were significant differences in metabolic phenotypes between the heartwood and sapwood in Shisham. The analysis of differential metabolites also showed that the expression patterns of metabolites in heartwood were similar, and they were significantly different from sapwood. Therefore, the analysis of different metabolites is helpful in understanding property differences between different tissues.

Firstly, the color of heartwood was darker than that of sapwood in this experiment, which may be due to the accumulation of extracts during the formation of heartwood. Some results show that the color-related compounds in wood, such as pigments, tannins, and resins, have phenolic hydroxyl, carbonyl, and double-bond structures [32]. However, the substance responsible for the coloring of heartwood is not clear. In a recent study, researchers speculated that 4-tert-butyl-2-phenyl-phenol, 2-methylanthraquinone, and 2,3-dimethyl-1,4,4a, 9a-tetrahydro-9,10-anthracene dione may be the primary compounds that lead to a darker heartwood color [33]. In our study, it was shown that various metabolites measured using UPLC-MS/MS, especially 105 different metabolites of heartwood and sapwood, may contain substances related to wood color characteristics, such as butein, kaempferol, dihydroglycitein, etc., which only exist in heartwood. Our metabolite data suggest the presence of new substances may affect the color of the heartwood, and these data provide a reference for future research on the color characteristics of *Dalbergia sissoo* wood.

The accumulation of extracts not only gives heartwood a deeper color, but also contributes greatly to the structural properties of wood. Previous studies on the biological activity and function of Dalbergia extracts mainly focused on insect resistance, antioxidation, and disease and insect resistance [34,35,36,37]. However, no systematic study has been carried out regarding the resistance mechanism and active substances of heartwood extracts, which can be aptly explained by the isolation and identification of wood extracts. In view of our experimental results, we speculate that flavonoids in heartwood play an important role in wood resistance. It is not only a chromogenic substance for plant tissues, but also acts as a plant defensin, protecting plants from biotic and abiotic stresses [38,39]. Flavonoids and other secondary metabolites have an important role in the process of plant defense against adversity, and the most significant role is involved in the detoxification, antioxidation, and ROS clearance of plants under adversity stress [40,41].

### 3.2. Differential Metabolites of Heartwood and Sapwood

Principal component analysis and the orthogonal partial least-squares method were used to analyze the samples; 105 differential metabolites were found between the two groups. Among them, the abundance of only 26 metabolites was higher in sapwood than in heartwood, accounting for 24.76%. Since the substance could not have been produced out of thin air, we discuss how a large number of secondary metabolites accumulated in the heartwood. Based on the description of Kampe and Magel [20], we summarize three production modes, including type I (Robinia pseudoacacia type), type II (walnut type), and type III (sandalwood type). Therefore, the detection of the location of secondary metabolites during xylem formation is helpful in determining the key clues that may explain heartwood formation. On this basis, we annotated the enrichment pathways of the differential metabolites between group X and group B. The results showed that most of the differential substances were annotated into three pathways: Flavonoid biosynthesis, isoflavonoid biosynthesis, and flavonoid and flavonol biosynthesis. Additionally, most of the detected substances were found to be in terminal position products. As far as our experimental results are concerned, it is presumed that the final formation pattern of secondary metabolites should belong to type I.

The results of the differential metabolite analysis showed that the levels of major primary metabolites or basic reaction substrates (such as citric acid and oxoglutaric acid) in sapwood were significantly higher than those in the heartwood. This result is consistent with previous studies regarding tissue anatomy and physicochemical properties [42]. Non-structural carbohydrates and lipids are energy storage substances in sapwood [43]. Previous studies regarding primary metabolites showed that the number of differential metabolites increased with an increase in distance from heartwood. We speculate that in the heartwood formation process, a large amount of energy storage substances is consumed in sapwood, which leads to the extremely low content of these energy storage substances in heartwood [44,45,46]. The formation of heartwood material is accompanied by the consumption of energy storage material, the disintegration of the nucleus, and the loss of chromatin, which eventually leads to the death of parenchyma cells [47,48].

Most of the differential metabolites in the experimental results were annotated to the biosynthesis of flavonoids. The biosynthesis of flavonoids is a complex regulatory network, that involves many vital genes and enzymes. It is well known that the biosynthetic pathway of flavonoids is derived from the phenylpropanoid pathway, which is derived from the shikimic acid pathway and acetic acid malonate pathway [49]. This series of metabolic pathways enables the formation of chalcones, anthocyanins, flavonoids, and others [50,51]. In our experiments, similar derivatives of substances in the process of flavonoid biosynthesis, such as naringenin 4′-o-glucuronide, P-chlorophenylalanine, and N-acetyl-d-phenylalanine, showed higher expression abundance in sapwood. Correspondingly, the end products of the synthetic pathway are primarily concentrated in heartwood. We speculate that the metabolic pathway of flavonoid biosynthesis proceeds with the transformation from sapwood to heartwood. The reason for the scarcity of intermediate substances in heartwood may be that the dead cells lack the corresponding enzymes to terminate the reaction or the end products of metabolism lead to cell death; these theories need to be verified.

## 4. Materials and Methods

### 4.1. Experimental Materials

The experimental tree was taken from the *Dalbergia sissoo* plantation in Yuanjiang experimental station, Institute of Highland Forest Science, Chinese Academy of Forestry, which was introduced from India. According to the growth situation of the existing *Dalbergia sissoo* plantation, six plants were randomly selected from the stand in December 2020 for analysis and determination, regardless of the effects of planting density and genetic differences.

After the trees were felled, wood discs were taken from the base of each stem (20 cm above the ground) and taken back to the laboratory to be frozen in liquid nitrogen, and then they were stored in a refrigerator at −80 °C until use. They were divided by color into heartwood (which formed group X) and sapwood (which formed group B). Samples were taken from the heartwood and sapwood portions at relatively appropriate locations, with 6 samples for each and a total of 12 samples. Then, the woodblocks of the two parts were cut into small sticks and freeze-dried. They were then ground into wood powder, and 40– -60-mesh wood powder was taken for use.

### 4.2. Sample Handling

The samples to be tested were processed according to the following steps [52]:

1. Accurately weigh 50 mg of sample into a 2 mL centrifuge tube and add a grinding bead with a diameter of 6 mm.
2. Add 400 μL of extraction solution (methanol: Water = 4:1 (V:V), containing 0.02 mg/mL of internal standard (l–2-chlorophenylalanine).
3. Grind for 6 min (−10 °C, 50 Hz) with a frozen tissue grinder.
4. Carry out low-temperature ultrasonic extraction for 30 min (5 °C, 40 kHz).
5. Leave the sample at −20 °C for 30 min.
6. Centrifuge for 15 min (13,000× *g*, 4 °C), and transfer the supernatant to the injection vial with an internal cannula for UPLC-MS/MS analysis.
7. In addition, take 20 μL of supernatant from each sample and mix it as the quality control sample (QC01, QC02, and QC03).

### 4.3. UPLC-MS/MS Conditions

Chromatographic separation of the metabolites was performed on a ExionLCTMAD system (AB Sciex, Milford, MA, USA) equipped with an ACQUITY UPLC BEH C18 column (100 mm × 2.1 mm i.d., 1.7 µm; Waters, Milford, MA, USA). The experimental conditions were set up in accordance with Yang [53]. The mobile phases consisted of 0.1% formic acid in water (solvent A) and 0.1% formic acid in acetonitrile:isopropanol (1:1, *v*/*v*) (solvent B). The solvent gradient changed according to Table 3. The sample injection volume was 20 μL, and the flow rate was set to 0.4 mL/min. The column temperature was maintained at 40 °C. During the period of analysis, all of these samples were stored at 4 °C.

The UPLC system was coupled to a quadrupole time-of-flight mass spectrometer (Triple TOFTM5600+, AB Sciex, Milford, MA, USA) equipped with an electrospray ionization (ESI) source operating in positive mode and negative mode. The following parameters were set in accordance with Yang [53]: Source temperature, 500 °C; curtain gas (CUR), 30 psi; both Ion Source GS1 and GS2, 50 psi; ion-spray voltage floating (ISVF), −4000 V in negative mode and 5000 V in positive mode, respectively; declustering potential, 80 V; collision energy (CE), 20–60 V rolling for MS/MS. Data acquisition was performed with the Data-Dependent Acquisition (DDA) mode. The detection was carried out over a mass range of 50–1000 *m*/*z*.

### 4.4. Data Preprocessing and Annotation

After UPLC-TOF/MS analyses, the raw data were imported into Progenesis QI 2.3 (Nonlinear Dynamics, Waters, Milford, MA, USA) for peak detection and alignment. The preprocessing was carried out in accordance with Guang [31]. The preprocessing results generated a data matrix consisting of the retention time (RT), mass-to-charge ratio (*m*/*z*) values, and peak intensity. Metabolic features detected at a level of at least 80% in any set of samples were retained. After filtering, minimum metabolite values were imputed for specific samples in which the metabolite levels fell below the lower limit of quantitation and each metabolic feature was normalized by sum. The internal standard was used for data QC (reproducibility), and metabolic features for which the relative standard deviation (RSD) of QC >30% were discarded. Following normalization procedures and imputation, statistical analysis was performed on log-transformed data to identify significant differences in metabolite levels between comparable groups. The mass spectra of these metabolic features were identified by using the accurate mass, and the MS/MS fragments spectra and isotope ratio difference were determined by searching in reliable biochemical databases such as the human metabolome database (HMDB) (http://www.hmdb.ca/, accessed on 15 March 2021) and the Metlin database (https://metlin.scripps.edu/, accessed on 16 March 2021).

### 4.5. Multivariate Statistical Analysis

Multivariate statistical analysis was performed using ropls (Version1.6.2, http://bioconductor.org/packages/release/bioc/html/ropls.html, accessed on 8 March 2021) R package from Bioconductor on the Majorbio Cloud Platform (https://cloud.majorbio.com, accessed on 8 March 2021). A PCA using an unsupervised method was applied to obtain and visualize an overview of the metabolic data, general clustering, trends, or outliers [54]. All metabolite variables were scaled to unit-variances prior to conducting the PCA. OPLS-DA was used for statistical analysis to determine global metabolic changes between the comparable groups. The metabolite variables were scaled to Pareto Scaling prior to conducting the OPLS-DA. The model validity was evaluated from the model parameters R2 and Q2. Variable importance in the projection (VIP) was calculated in the OPLS-DA model. The *p*-values were estimated with a paired Student’s *t*-test on single-dimensional statistical analysis.

Metabolites (VIP >1.0) were assumed to be vital metabolites for the potential discrimination of samples in the OPLS-DA models. Databases were used to analyze metabolite pathways, including the KEGG (Kyoto Encyclopedia of Genes and Genomes, http://www.genome.jp/kegg/, accessed on 28 March 2021) database, to identify affected metabolic pathways and facilitate further metabolite interpretation.

## 5. Conclusions

Although rosewood has essential economic and ecological value, little is known about the regulatory mechanism of heartwood formation. In this paper, based on UPLC-MS/MS, the first metabolomic analysis of the heartwood and sapwood of Shisham stem was carried out. A total of 735 metabolites were identified, among which the largest number of terpenoid species and the highest content of flavonoids were found. The analysis of differential metabolites in heartwood and sapwood showed that the content level of organic oxygen compounds and flavonoids was higher. Analysis of 105 differential metabolites showed that only 26 species were more abundantly expressed in sapwood than in heartwood. Through metabolic pathway analysis, we found that the metabolites with higher expression in sapwood were mostly located upstream of the metabolism, and most of them belonged to primary metabolites. In contrast, most of the enriched substances in heartwood were located downstream of the metabolic pathway. The differential metabolites were mainly enriched in three metabolic pathways: Flavonoid biosynthesis, isoflavonoid biosynthesis, and flavone and flavonol biosynthesis. The role of differential metabolites in heartwood color and wood resistance was reasonably well discussed. This study provides a basis for future studies on heartwood formation in *Dalbergia* and other woody plants and is of great theoretical reference importance.

## Figures and Tables

**Table 1:** Total ion count and identification statistics.

Ion Mode	All Peaks	Identified Metabolites	Metabolites in Library	Metabolites in KEGG
pos	5704	346	311	94
neg	4894	389	365	88

**Table 2:** Enrichment analysis of metabolic pathways of differential metabolites.

Pathway ID	Description	Number	Metabolites	Retention Time	Class
map00941	Flavonoid biosynthesis	7	Butein	3.833	Chalcones
			Pinocembrin	5.7929	Flavanones
			Hesperetin	4.5791	Flavanones
			Dihydrokaempferol	4.1904	Dihyroflavonols
			Kaempferol	3.554	Flavonols
			Pelargonidin	3.87	Anthocyanidins and anthocyanins
			Cyanidin	3.5594	Anthocyanidins and anthocyanins
map00943	Isoflavonoid biosynthesis	4	Daidzein	4.2676	Isoflavones
			Formononetin	5.2318	Isoflavones
			Glycitein	4.1901	Isoflavones
			Genistein	4.2578	Isoflavones
map00944	Flavone and flavonol biosynthesis	2	Kaempferol	3.554	Flavonols
			Acacetin	4.3609	Flavones

**Table 3:** Time variation of the solvent gradient.

Time (min)	A (%)	B (%)
0	95	5
3	80	20
9	5	95
13	5	95
13.1	95	5
16	95	5

## Data Availability

Not available.

## Supplementary Materials

The supplementary materials are available at https://www.mdpi.com/article/10.3390/molecules27061982/s1, Table S1: The results of the positive ion table; Table S2: The results of the negative ion table; Table S3: A list of the 735 metabolites detected in this study.

## Sample Availability

Samples of the compounds are not available from the authors.
